# Development of a pan-Simbu real-time reverse transcriptase PCR for the detection of Simbu serogroup viruses and comparison with SBV diagnostic PCR systems

**DOI:** 10.1186/1743-422X-10-327

**Published:** 2013-11-05

**Authors:** Melina Fischer, Horst Schirrmeier, Kerstin Wernike, Anne Wegelt, Martin Beer, Bernd Hoffmann

**Affiliations:** 1 Institute of Diagnostic Virology, Friedrich-Loeffler-Institut, Südufer 10, D-17493, Greifswald-Insel Riems, Germany

**Keywords:** Orthobunyavirus, Simbu serogroup, Schmallenberg virus, Real-time RT-PCR

## Abstract

**Background:**

Schmallenberg virus (SBV), a novel orthobunyavirus of the Simbu serogroup, was first identified in October 2011 in dairy cattle in Germany, where it caused fever, diarrhea and a drop in milk yield. Since then, SBV additionally has been detected in adult sheep and goats. Although symptoms of acute infection were not observed, infection during a vulnerable phase of pregnancy caused congenital malformations and stillbirths. In view of the current situation and the possible emergence of further Simbu serogroup members, a pan-Simbu real-time reverse transcriptase (RT) PCR system for the reliable detection of Simbu serogroup viruses should be developed.

**Methods:**

In this study a pan-Simbu real-time RT-PCR system was established and compared to several SBV real-time RT-PCR assays. All PCR-systems were tested using a panel of different Simbu serogroup viruses as well as several field samples from diseased cattle, sheep and goats originating from all over Germany. Several pan-Simbu real-time RT-PCR products were sequenced via Sanger sequencing. Furthermore, *in silico* analyses were performed to investigate suitability for the detection of further orthobunyaviruses.

**Results:**

All tested members of the Simbu serogroup (n = 14) as well as most of the field samples were successfully detected by the pan-Simbu real-time RT-PCR system. The comparison of this intercalating dye assay with different TaqMan probe-based assays developed for SBV diagnostics confirmed the functionality of the pan-Simbu assay for screening purposes. However, the SBV-TaqMan-assay SBV-S3 delivered the highest analytical sensitivity of less than ten copies per reaction for duplex systems including an internal control. In addition, for confirmation of SBV-genome detection the highly specific SBV-M1 assay was established.

**Conclusion:**

The pan-Simbu real-time RT-PCR system was able to detect all tested members of the Simbu serogroup, most of the SBV field samples as well as three tested Bunyamwera serogroup viruses with a suitable sensitivity. According to *in silico* analyses, this system seems to be able to detect a broad orthobunyavirus spectrum. As an additional feature of the pan-Simbu real-time RT-PCR system, subsequent species classification via sequencing is feasible. Regarding SBV diagnostics, the performance of the S-segment targeting SBV-S3 assay was superior with respect to the analytical sensitivity.

## Background

In October 2011, a novel orthobunyavirus (family: *Bunyaviridae*) related to the Simbu serogroup viruses affected dairy cattle on a farm near the city of Schmallenberg (North Rhine-Westphalia, Germany). The virus caused clinical signs such as fever, diarrhea and decreased milk yield in dairy cattle. It was finally identified via full-genome sequencing combined with metagenome analysis and named Schmallenberg virus (SBV) after its geographical origin [1]. Subsequently, SBV was detected in samples from diseased cattle in the Netherlands and in malformed sheep and goat lambs as well as in calves from several European countries [2].

Orthobunyaviruses are characterized by a tripartite, single-stranded, negative-sense RNA genome [3,4]. The three segments are termed according to their size as small (S), medium (M) and large (L) [5]. The genus is divided into 18 serogroups defined by serological relationships. One of the largest serogroups, the Simbu serogroup, comprises at least 25 members [6]. Full-genome sequencing as well as Sanger sequencing and subsequent phylogenetic analyses revealed that SBV is a Simbu serogroup virus, closely related to viruses of the species *Sathuperi virus*[7]. Sequencing also revealed a complementarity of the 3 prime and the 5 prime end of each segment, a typical feature of the members of the family *Bunyaviridae,* which leads to the formation of panhandle structures [8].

In this study a pan-Simbu real-time reverse transcriptase (RT) PCR system was developed based on two conserved sequence regions of the L-segment facilitating the amplification of a 279 base pair (bp) fragment for the reliable detection of viruses predominantly from the Simbu serogroup. Furthermore, several diagnostic real-time RT-PCR assays for the sensitive and specific detection of the SBV genome were developed and validated. Within the SBV L-segment the SBV-L1 and the SBV-L1.4 assays detected an amplification fragment of 144 bp and 107 bp in length, respectively. The SBV-M1 assay produced a 137 bp PCR product from the M-segment, whereas the published SBV-S3 assay detected an 88 bp region of the S-segment [9]. All of these PCR-systems were tested with a panel of different Simbu serogroup viruses as well as several SBV field samples from diseased cattle, sheep and goats originating from all over Germany.

## Results and discussion

**Figure 1:**
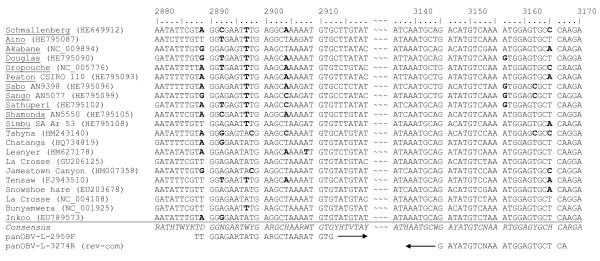
**Comparison of the consensus sequences of different *Orthobunyavirus *species.** Sequences are in regard to the primer region of the L-segment. The nucleotide positions are based on the Schmallenberg virus genome [GenBank: HE649912]. Letters in bold represent nucleotides with mismatch to the primer sequence. Simbu serogroup viruses are underlined.

As a first step, from our perspective pivotal members of the Simbu serogroup (Aino virus AINOV, Akabane virus AKAV, Douglas virus DOUV, Oropouche virus OROV, Peaton virus PEAV, Sabo virus SABOV, Sango virus SANV, Sathuperi virus SATV, Shamonda virus SHAV, Shuni virus SHUV, Simbu virus SIMV, Thimiri virus THIV, Tinaroo virus TINV; see Table 1 for species classification) including SBV and three members of the Bunyamwera serogroup (Batai virus BATV, Bunyamwera virus BUNV, Ngari virus NRIV) were tested to define the detection spectrum of the intercalating dye based pan-Simbu real-time RT-PCR system and the TaqMan probe-based SBV real-time RT-PCR assays (Table 1). Although the applied virus titers were partially low (1 × 10^2^, 1 × 10^2.5^ or 1 × 10^3^ 50% tissue culture infectious doses (TCID_50_) per ml), the pan-Simbu real-time RT-PCR system detected all tested isolates of the Simbu serogroup. DOUV however was recognized with a high quantification cycle (C_q_) value of 35.7 presumably caused by mismatches in the binding region of the forward primer (Figure 1). Also the detection of THIV seems problematic, yielding the highest C_q_-value (36.2) in this investigation. To further characterize the pan-Simbu real-time RT-PCR system the limit of detection and the PCR efficiency were determined using an SBV L-segment standard. The assay reliably detected 100 copies per reaction and displayed an efficiency of 91.6%.

**Table 1:** PCR analysis of Simbu serogroup viruses and Bunyamwera serogroup viruses

				**Pan Simbu**	**SBV assays**			
Species*	**Virus**	**Strain**	**TCID**_**50**_**/ml**		**M1**	**L1**	**L1.4**	**S3**
Akabane	**AKAV**	OBE-1	1 × 10^5.4^	33.7	**neg.**	**neg.**	**neg.**	**neg.**
Akabane	**SABOV**	AN 9398	1 × 10^2.5^	29.3	**neg.**	**neg.**	**neg.**	**neg.**
Akabane	**TINV**	CSIRO 153	1 × 10^3.1^	30.2	**neg.**	**neg.**	**neg.**	**neg.**
Bunyamwera	**BATV**	53.2	1 × 10^5^	32.7	**neg.**	**neg.**	**neg.**	**neg.**
Bunyamwera	**BUNV**^**#**^	ATCC VR-87	1× 10^4.5^	33.1	**neg.**	**neg.**	**neg.**	**neg.**
Bunyamwera	**NRIV**^**#**^	51	-	31.7	**neg.**	**neg.**	**neg.**	**neg.**
Oropouche	**OROV**	TRVL 9760	1 × 10^3.5^	33.2	**neg.**	**neg.**	**neg.**	**neg.**
Sathuperi	**SATV**	l-11155	1 × 10^2^	28.5	**neg.**	**neg.**	**neg.**	**neg.**
Sathuperi	**DOUV**	CSIRO 150	1 × 10^2.5^	35.7	**neg.**	**neg.**	**neg.**	25.4
Sathuperi	**SBV**	BH80-11	1 × 10^4.3^	30.6	22.5	23.3	23.6	21.9
Shamonda	**SHAV**	An 5550	1 × 10^2^	30.6	**neg.**	30.0	23.9	22.2
Shamonda	**PEAV**	CSIRO 110	1 × 10^2^	31.4	**neg.**	**neg.**	**neg.**	**neg.**
Shamonda	**SANV**	An 5077	1 × 10^3^	30.1	**neg.**	**neg.**	**neg.**	**neg.**
Shuni	**SHUV**	An 10107	1 × 10^2.5^	28.0	**neg.**	**neg.**	**neg.**	**neg.**
Shuni	**AINOV**	Ja N Ar 28	1 × 10^2^	27.4	**neg.**	**neg.**	**neg.**	**neg.**
Simbu	**SIMV**	SA Ar 53	1 × 10^3^	27.2	**neg.**	**neg.**	**neg.**	**neg.**
Thimiri	**THIV**	-	1 × 10^4.1^	36.2	**neg.**	**neg.**	**neg.**	**neg.**

**neg.:** negative result; -: not available; pan Simbu: pan-Simbu real-time RT-PCR system; TCID_50_: 50% tissue culture infectious doses; M1: highly specific SBV-M1 assay; L1: SBV-L1 assay, L1.4: SBV-L1.4 assay; S3: SBV-S3 assay; *species classification according to [10], SBV [7]; ^#^1:100 dilution of original RNA; columns 5–9 represent mean C_q_-values from duplicates.

The potential of a subsequent species classification via sequencing was investigated for AINOV, DOUV, PEAV, SABOV, SANV, SATV, SHAV, SHUV and SIMV. Each sequence fragment displayed 100% sequence identity with the respective Simbu serogroup virus in GenBank, whereas 60-88% sequence identity was determined among the amplified products, facilitating a reliable differentiation. *In silico* analyses indicated that this pan-Simbu system is probably able to detect also members from other serogroups, e.g. several members of the California serogroup: Tahyna-, Chatanga-, La Crosse-, Jamestown Canyon-, Snowshoe hare- and Inkoo virus [11] (Figure 1). This assumption was supported by detection of the Bunyamwera serogroup viruses BATV, BUNV and NRIV (Table 1). However, it cannot be excluded that some untested members could be only poorly or not detected at all. Previously published real-time RT-PCR systems are available e.g. for the specific detection of OROV [12] as well as for the simultaneous detection of AKAV and AINOV [13], but to our knowledge this pan-Simbu PCR is the first generic real-time PCR approach for the detection of orthobunyaviruses, predominantly members of the Simbu serogroup.

The different Simbu serogroup viruses and the three Bunyamwera serogroup viruses were also analyzed with SBV-specific real-time RT-PCR assays. With this approach, only the SBV-M1 assay scored negative for the Bunyamwera serogroup viruses and for all Simbu serogroup viruses, with the exception of SBV (Table 1). This assay seemed to be highly specific for SBV. In addition to SBV, the SBV-L1 and the SBV-L1.4 assays also recognized RNA from a SHAV (species *Shamonda virus*, reassortant comprising the SBV S- and L-segments and the M-segment from an unclassified virus [7]) sample. Compared to each other, these assays displayed markedly different C_q_-values for the detection of SHAV presumably due to differences in the primer binding capability caused by a slight adjustment of the detection region for the SBV-L1.4 assay. The SBV-S3 assay was able to detect RNA from SBV, SHAV and DOUV (species *Sathuperi virus*; see Table 1 for species classification). In this case, the detection region of the SBV-S3 assay on the S-segment is highly similar between SBV and the reassortant SHAV and further seems to be rather similar between the two members of the *Sathuperi virus* species SBV and DOUV which consequently enables their recognition.

To evaluate the sensitivity of the different assays a log_10_ dilution series (10^-2^ to 10^-8^ dilution) of SBV-RNA extracted from a cell culture sample (BH80/11-4, initial titer 1 × 10^6.3^ TCID_50_/ml) was tested (Table 2). The maximum detection rate of the pan-Simbu real-time RT-PCR system was seen at the 10^-4^ dilution; due to recognition up to this dilution step, the system seems sufficient for a preliminary screening of undiluted field samples of good quality. The SBV-S3 assay provided the best performance with a reliable recognition (detection of duplicate samples) of every dilution step up to the 10^-7^ dilution in combination with the lowest C_q_-values compared to the other assays. Furthermore, the SBV-L1 and SBV-L1.4 assays displayed comparable C_q_-values for the dilution series with a reliable detection up to a dilution of 10^-6^. The SBV-specific SBV-M1 assay allowed a positive detection up to the dilution step 10^-5^. Despite its lower sensitivity, the SBV-M1 assay is a valuable tool for distinguishing SBV from other orthobunyaviruses to yield a precise diagnosis in regions where multiple orthobunyaviruses occur.

**Table 2:** Evaluation of the sensitivity of the individual assays using an SBV-RNA dilution series

	**Pan Simbu**	**SBV assays**			
Sample*		**M1**	**L1**	**L1.4**	**S3**
SBV 10^-2^	30.6	22.5	23.3	23.6	21.9
SBV 10^-3^	34.5	26.1	26.8	26.6	24.5
SBV 10^-4^	38.2	29.2	30.1	30.1	27.8
SBV 10^-5^	**neg.**	32.6	33.3	33.3	31.1
SBV 10^-6^	**neg.**	41.1^#^	36.4	36.8	34.6
SBV 10^-7^	**neg.**	**neg.**	**neg.**	39.6^#^	37.8
SBV 10^-8^	**neg.**	**neg.**	**neg.**	**neg.**	**neg.**

*log10 dilution series of SBV RNA extracted from cell culture sample BH80/11-4 (KC/BHK), initial titer 1× 10^6.3^ TCID50/ml; ^#^only one of the duplicates positive; **neg.**: negative result; pan Simbu: pan-Simbu real-time RT-PCR system; M1: SBV-M1 assay; L1: SBV-L1 assay, L1.4: SBV-L1.4 assay; S3: SBV-S3 assay; columns 2–6 represent mean C_q_-values from duplicates.

In addition to the sensitivity analysis, the pan-Simbu real-time RT-PCR system, the SBV-M1, the SBV-L1, the SBV-L1.4 and the SBV-S3 assays were tested using 47 RNA samples from SBV-suspicious field material as well as a defined sample from an SBV challenge experiment [1] (Table 3). In this approach, the SBV-S3 assay showed the lowest C_q_-values among the tested assays and recognized 40 positive samples, while eight isolates scored negative (pre-tested as negative by SBV-L1 assay).

**Table 3:** Comparison of pan-Simbu real-time RT-PCR and SBV diagnostic PCRs using field samples

				**Pan Simbu**	**SBV assays**			
**Sample**	**Material**	**Species**	**Origin**		**M1**	**L1**	**L1.4**	**S3**
1	Brain	Sheep (lamb)	NRW	34.1	27.2	27.2	26.7	23.7
2	Cerebellum	Goat (goatling)	NRW	32.3	26.3	25.5	26.3	22.5
3	Med. obl.	Goat (goatling)	NRW	36.0	31.0	28.3	29.1	25.5
4	Brain	Goat (goatling)	NRW	34.4	28.3	24.5	24.9	21.5
5	Inter. Candolis	Goat (goatling)	NRW	33.2	27.3	25.5	26.2	22.6
6	Brain	Sheep (fetus)	NRW	39.3	38.2	37.8	39.4	33.9
7	Blood	Sheep (lamb)	NRW	38.1	36.6	35.4	36.0	32.2
8	Med. obl.	Sheep (lamb)	NRW	35.4	28.0	29.9	30.8	27.1
9	Cerebellum	Sheep (lamb)	NRW	38.3	34.8	34.3	34.7	31.7
10	Cerebrum	Sheep (lamb)	NRW	37.0	32.6	32.2	33.0	31.4
11	Spinal cord	Sheep (lamb)	NRW	36.7	30.5	30.6	31.7	28.1
12	Blood	Sheep (lamb)	NRW	**neg.**	32.0	**neg.**	36.1	31.9
13	Med. obl.	Sheep (lamb)	NRW	37.2	32.6	32.1	33.0	28.3
14	Cerebellum	Sheep (lamb)	NRW	38.7	31.4	33.8	35.5	30.4
15	Cerebrum	Sheep (lamb)	NRW	36.6	30.7	29.9	30.5	26.3
16	Cerebrum	Sheep (lamb)	NRW	32.6	27.0	25.4	26.3	23.1
17	Med. obl.	Sheep (lamb)	NRW	39.0	34.2	37.2	38.6	32.3
18	Cerebrum	Sheep (lamb)	NRW	29.7	22.6	22.5	21.3	16.8
19	Cerebrum	Sheep (lamb)	NRW	39.9*	**neg.**	**neg.**	39.2*	37.0
20	EDTA blood	Sheep	Hesse	38.8*	34.8	34.5	34.7	30.3
21	EDTA blood	Sheep	Hesse	**neg.**	38.6	37.4	37.3	34.7
22	Organ material	Sheep	Hesse	28.9	25.5	20.3	20.1	15.2
23	Cerebrum	Sheep (lamb)	Hesse	38.1	29.8	31.0	31.7	28.1
24	Brain stem	Sheep	LS	36.4	32.4	28.7	29.3	26.4
25	Cortex	Sheep	LS	36.5	33.2	30.1	30.7	27.5
26	Brain	Sheep (lamb)	LS	38.0	32.9	31.3	31.1	28.6
27	Brain	Goat (goatling)	BW	31.9	25.8	21.7	22.1	18.1
28	Serum	Goat (goatling)	BW	39.6*	35.5	32.0	32.4	28.8
29	Cruor	Goat (goatling)	BW	34.1	30.2	28.5	29.0	25.5
30	Cerebellum	Sheep	SA	34.1	31.1	30.6	30.4	28.0
31	Cerebrum	Sheep	SA	36.7	32.7	31.7	31.8	29.2
32	Cerebrum	Sheep	SA	29.8	25.3	22.2	22.4	21.8
33	Brain	Sheep (fetus)	Bavaria	33.7	30.2	25.0	24.6	22.0
34	Brain	Sheep (fetus)	Bavaria	**neg.**	33.8	36.0	36.7	32.9
35	EDTA blood	Sheep (fetus)	Bavaria	**neg.**	36.2	**neg.**	38.7*	34.9
36	Organ material	Sheep (lamb)	HH	**neg.**	**neg.**	**neg.**	**neg.**	**neg.**
37	Organ material	Sheep	HH	36.5	26.4	27.5	27.3	25.4
38	Organ material	Sheep (lamb)	SH	38.9	32.9	31.2	30.7	27.7
39	Serum	Cattle	TH	**neg.**	**neg.**	**neg.**	**neg.**	**neg.**
40	Cerebrum	Bison (fetus)	RP	29.4	22.2	21.1	21.6	15.8
41	Cerebrum	Sheep	BB	**neg.**	33.6	32.3	32.7	28.2
42	Blood	Cattle	animal trial	29.7	20.4	21.9	22.9	21.1
43	Blood	Sheep (lamb)	NRW	**neg.**	**neg.**	**neg.**	**neg.**	**neg.**
44	Med. obl.	Sheep (lamb)	NRW	**neg.**	**neg.**	**neg.**	**neg.**	**neg.**
45	Cerebellum	Sheep (lamb)	NRW	**neg.**	**neg.**	**neg.**	**neg.**	**neg.**
46	Cerebrum	Sheep (lamb)	NRW	**neg.**	**neg.**	**neg.**	**neg.**	**neg.**
47	Spleen	Sheep (lamb)	NRW	**neg.**	**neg.**	**neg.**	**neg.**	**neg.**
48	Muscle	Sheep (lamb)	NRW	**neg.**	**neg.**	**neg.**	**neg.**	**neg.**

med. obl.: medulla oblongata; organ material consists of cerebrum, cerebellum, spleen; LS: Lower Saxony; NRW: North Rhine-Westphalia; SH: Schleswig-Holstein; TH: Thuringia; RP: Rhineland-Palatinate; BW: Baden-Württemberg; HH: Hamburg; BB: Brandenburg; SA: Saxony-Anhalt; * only one of the duplicates positive; **neg.**: negative result; pan Simbu: pan-Simbu real-time RT-PCR system; M1: SBV-M1 assay; L1: SBV-L1 assay, L1.4: SBV-L1.4 assay; S3: SBV-S3 assay; columns 5–9 represent mean C_q_-values from duplicates.

The pan-Simbu real-time RT-PCR system detected 32 positive samples out of the 40 SBV-S3-positive samples. Three samples which had C_q_-values above 28 using the SBV-S3 assay scored doubtful with only one positive duplicate using the pan-Simbu real-time RT-PCR system. Eight samples defined as SBV-negative with the SBV-S3 assay, and additional five samples with a genome load of C_q_-values >28 using the SBV-S3 assay, scored negative in the pan-Simbu real-time RT-PCR system. In general, the pan-Simbu real-time RT-PCR system produced higher C_q_-values than the SBV-specific assays (Table 3). Therefore, the sensitivity of the pan-Simbu system presumably was not sufficient for samples containing a low viral genome load. Nevertheless, the pan-Simbu real-time RT-PCR system was able to detect field samples with C_q_-values below 28 reliably measured by the SBV-S3 assay as well as the animal trial sample (Table 3).

Furthermore, the SBV-M1 assay revealed 39 positive and nine negative samples (Table 3). One SBV-S3 positive sample (C_q_ 37) scored negative, presumably due to the reduced sensitivity of the SBV-M1 assay. The SBV-L1 assay detected 37 positive and eleven negative samples. Three samples tested positive with C_q_-values >31 in the SBV-S3 assay scored negative. The SBV-L1.4 assay yielded comparable C_q_-values to the SBV-L1 assay, but recognized 38 positive samples, and two doubtful results (SBV-S3 C_q_-values >34) with only one positive duplicate displaying a Cq-value higher than 38. Eight isolates scored negative (in concordance with SBV-S3) with both SBV-L assays.

Finally, the analytical sensitivity of the SBV-S3 single-target assay in comparison to two different SBV-S3 duplex assays including internal amplification controls was evaluated (Table 4). The single-target assay detected a dilution of 2 × 10^-1^ copies/μl, which corresponded to one RNA copy per reaction. Both duplex assays were able to detect at most 2 × 10^0^ copies/μl (10 copies per reaction), while simultaneous co-amplification of either IC2-RNA (~C_q_ 26) or the housekeeping gene beta-actin (~C_q_ 29) was performed reliably.

**Table 4:** Evaluation of the analytical sensitivity of the SBV-S3 assay using an SBV-S3 standard

	**Viral genome**			**Internal control**	
**Concentration**	**Single**	**Duplex IC2**	**Duplex β-actin**	**Duplex IC2**	**Duplex β-actin**
2 × 10^6^	16.3	16.6	16.5	26.7	29.4
2 × 10^5^	19.5	19.8	19.8	26.2	28.5
2 × 10^4^	23.0	23.1	23.3	26.1	28.3
2 × 10^3^	26.3	26.4	26.5	26.2	28.2
2 × 10^2^	29.6	29.8	30.0	26.2	28.3
2 × 10^1^	32.5	32.7	33.6	26.3	28.4
2 × 10^0^	34.4	34.8	34.6	26.4	28.5
2 × 10^-1^	37.6	39.3*	**neg.**	26.4	28.7

concentration in copies/μl; single: SBV-S3 single-target assay; duplex IC2: SBV-S3 duplex assay based on IC2 RNA; duplex β-actin: SBV-S3 duplex assay with co-amplification of beta-actin gene; *only one of the duplicates positive; **neg.**: negative result; columns 2–6 represent mean C_q_-values from duplicates.

## Conclusion

In conclusion, the pan-Simbu real-time RT-PCR system was able to detect all tested members of the Simbu serogroup as well as most of the SBV field samples and three Bunyamwera serogroup viruses with an acceptable sensitivity for screening diagnostics. Although we were not able to test all 25 members of the Simbu serogroup, we investigated key-members with clinical or economic importance like OROV and AKAV [6]. According to *in silico* analyses the system seems to be able to detect a broad spectrum of orthobunyaviruses (Figure 1) as supported by detection of members from the Bunyamwera serogroup (Table 1). The pan-Simbu real-time RT-PCR system is based on two highly conserved sequence regions of the L-segment which were selected as primer binding sites. As an additional feature of the novel system, these primers enclosed a variable sequence region, which is suitable for subsequent species classification via sequencing, avoiding misdiagnosis. This opportunity was confirmed exemplarily for AINOV, DOUV, PEAV, SABOV, SANV, SATV, SHAV, SHUV and SIMV. The here described pan-Simbu PCR system is therefore the first available tool for a broad screening of samples predominantly for Simbu serogroup virus genomes and could also allow the identification of related orthobunyaviruses in mammalian but also insect vector samples.

For the specific detection of SBV-genome, the SBV-S3 assay turned out to be the most suitable system with the highest sensitivity and reliability. The analytical sensitivity of the SBV-S3 single-target assay as well as the two duplex assays including an internal amplification control (IC2-RNA, beta-actin respectively) were determined as one copy per reaction performing the single-target assay and ten copies per reaction using both duplex systems. Furthermore, the SBV-S3 assay was also able to detect the highly related *Sathuperi virus* species member DOUV as well as the reassortant SHAV apart from SBV.

## Methods

### Samples and RNA isolation

Viral RNA from field samples, from blood of an experimentally infected calf [1] (Table 3) as well as from cell culture material of the Simbu serogroup viruses namely AINOV, AKAV, DOUV, OROV, PEAV, SABOV, SANV, SATV, SHAV, SHUV, SIMV, THIV, TINV [6] and from the Bunyamwera serogroup viruses BATV [14], BUNV, NRIV was extracted manually using the QIAmp Viral RNA Mini Kit (Qiagen, Hilden, Germany) according to the manufacturer’s instructions or using the MagNA Pure LC 2.0 in combination with the MagNA Pure LC Total Nucleic Acid Isolation Kit (Roche Diagnostics, Mannheim, Germany) according to the manufacturer’s recommendations. All samples were stored at -20°C until use.

### Oligonucleotides and primer design

The sequences of primers and probes are listed in Table 5. For the pan-Simbu assay, primers were selected by analyzing the consensus sequence of the L-segment of 70 orthobunyaviruses available in GenBank. The locations of the primers and potential mismatches with the L-segment of different orthobunyaviruses are depicted in Figure 1.

**Table 5:** Oligonucleotides used in this study

**Assay**	**Name**	**Oligonucleotide**	**Position (nt)**	**Sequence (5′-3′)**	**Location**
Pan-Simbu PCR	panOBV-L-2959 F	Primer	2888-3167	TTG GAG ART ATG ARG CTA ARA TGT G	L-Segment
	panOBV-L-3274R	Primer		TGA GCA CTC CAT TTN GAC ATR TC	
SBV-M1	SBV-M1-213 F	Primer	1690-1827	TCA ATT CAG CAA GTA ACA TAC AAT GG	M-Segment
	SBV-M1-350R	Primer		CGT GGT CTG TCT TGG TTG ATG	
	SBV-M1-240FAM	Probe		FAM-AAG CAC TGG CCC GAA GTT TCA CCT-BHQ1	
SBV-L1	SBV-L1-11 F	Primer	367-511	TTG CCG TTT GAT TTT GAA GTT GTG	L-Segment
	SBV-L1-155R	Primer		TCA GGG ATC GCA AAT TAA AGA ACC	
	SBV-L1-36FAM	Probe		FAM-TCA TCC GTG CTG ACC CTC TGC GAG-BHQ1	
SBV-L1.4	SBV-L1.2 F	Primer	361-468	TCA GAA TTG CCG TTT GAT TTT GAA G	L-Segment
	SBV-L1.4R	Primer		GTT GAG CGG CCC AAA TAT TTC C	
	SBV-L1-36FAM	Probe		FAM-TCA TCC GTG CTG ACC CTC TGC GAG-BHQ1	
SBV-S3*	SBV-S-382 F	Primer	382-469	TCA GAT TGT CAT GCC CCT TGC	S-Segment
	SBV-S-469R	Primer		TTC GGC CCC AGG TGC AAA TC	
	SBV-S-408FAM	Probe		FAM-TTA AGG GAT GCA CCT GGG CCG ATG GT-BHQ1	

N: any base; R: purine base; FAM: 6-carboxyfluorescein; HEX: 5′-hexachlorofluorescein; BHQ1: black hole quencher 1; positions according to [1] accession numbers [GenBank: HE649912-HE649914]; * protocol according to [9].

The selection of primers and probes of the SBV real-time RT-PCR assays was based on the first available sequence data of the SBV isolate BH80/11-4 [1] and was supported by the software package Beacon Designer 7.0 (PremierBiosoft, Palo Alto, USA).

All oligonucleotides were synthesized by metabion (metabion international AG, Martinsried, Germany) and stored at -20°C until use.

### Pan-Simbu real-time RT-PCR

In order to allow a broad detection of members of the Simbu serogroup a pan-Simbu real-time RT-PCR was developed using the OneStep RT-PCR Kit (Qiagen, Hilden, Germany). The assay was optimized using a total reaction volume of 25 μl. For one reaction 8 μl RNase-free water, 5 μl 5x OneStep RT-PCR Buffer, 1 μl OneStep RT-PCR Enzyme Mix, 1 μl dNTP Mix (10 mM each), 1 μl ResoLight Dye (Roche, Mannheim, Germany), 2 μl of each primer (panOBV-L-2959 F, panOBV-L-3274R; 10 μM each; Table 5) and 5 μl RNA template or RNase free water for the no template control (NTC) was used. The following thermal program was applied 1 cycle of 50°C for 30 min and 95°C for 15 min, followed by 40 cycles of 95°C for 30 s, 55°C for 30 s, 72°C for 30 s and 78°C for 15 s. The collection of the fluorescence data was performed during the 78°C elongation step.

### PCR efficiency and limit of detection for pan-Simbu real-time RT-PCR

The PCR efficiency and the limit of detection for the pan-Simbu real-time RT-PCR were determined using an SBV L-segment plasmid (L-segment sequence according to GenBank: HE649912 in X8δT backbone). The exact number of plasmid molecules was calculated (http://www.molbiol.edu.ru/eng/scripts/01_07.html) and a log_10_ dilution series (2 × 10^6^ to 2 × 10^-1^ copies/μl) was prepared. This dilution series was tested with the pan-Simbu real-time RT-PCR in four replicates. PCR efficiency was calculated by the CFX manager software 3.0 (Bio-Rad Laboratories Inc., Hercules, USA).

### Sequencing of pan-Simbu real-time RT-PCR products

For species classification of members of the Simbu serogroup (i.e. AINOV, DOUV, PEAV, SABOV, SANV, SATV, SHAV, SHUV and SIMV) obtained PCR products of the pan-Simbu real-time RT-PCR were analyzed on a 1.5% agarose gel and purified using the QIAquick Gel Extraction Kit (Qiagen, Hilden, Germany) according to the manufacturer’s instructions. Direct sequencing was carried out in both directions by termination cycle sequencing using the Big Dye Terminator Mix 1.1 (Applied Biosystems, Carlsbad, USA) with the same primers used for PCR product amplification. The assay was optimized by using a total reaction volume of 10 μl. Briefly, for one reaction 1 μl RNase-free water, 1 μl of 5x Sequencing Buffer, 2 μl Big Dye Terminator Mix 1.1 and 1 μl of the according primer (5 μM). The following thermal program was applied 1 cycle of 96°C for 1 min followed by 26 cycles of 95°C for 15 s, 53°C for 10 s, and 60°C for 4 min. After that, cycle sequencing products were purified with DyeEx 2.0 Spin Kit (Qiagen, Hilden, Germany). The nucleotide sequences were resolved in an ABI 3130 Genetic Analyzer (Applied Biosystems, Carlsbad, USA). Sequences were aligned and analyzed in BioEdit [15] (version 7.1.7).

### SBV-specific real-time RT-PCR assays

For detection of SBV-RNA several diagnostic assays (named SBV-M1, SBV-L1, SBV-L1.4 and SBV-S3) were tested using the AgPath-ID™ One-Step RT-PCR Kit (Applied Biosystems, Carlsbad, USA). The assays were optimized using a total reaction volume of 25 μl. Briefly, for one single reaction 4.5 μl RNase-free water, 12.5 μl 2× RT-PCR buffer, 1 μl 25× RT-PCR enzyme mix, 5 μl RNA template or RNase free water for the no template control (NTC) and 2 μl of the according primer-probe mix (Table 5) were combined. Based on stock solutions of the primer and probes of 100 pmol/μl (100 μM), the different primer-probe mixes were created. For each assay 20 μl of the relevant forward and reverse primer, 3.75 μl of the 6-carboxyfluorescein (FAM) labeled probe were mixed in 156.25 μl 0.1× Tris-EDTA (TE) buffer. Thus, a final concentration of 10 μM for each primer and 1.88 μM for each probe was used. The following thermal program was applied: 1 cycle of 45°C for 10 min and 95°C for 10 min, followed by 45 cycles of 95°C for 15 s, 56°C for 20 s, and 72°C for 30 s.

### Generation of a SBV-S3 PCR standard and identification of the analytical sensitivity

In order to determine the analytical sensitivity of the SBV-S3 assay, an SBV-S3 standard was generated. Therefore, the gel-purified SBV-S3 PCR fragment was ligated into the vector pGEM-Teasy (Promega, Mannheim, Germany). Plasmids were amplified in *Escherichia coli* DH10B (Invitrogen, Carlsbad, USA) and purified by Qiagen Plasmid Mini and Midi Kits (Qiagen, Hilden, Germany) according to standard protocols. The identity of the plasmids was confirmed by *EcoR*I-digestion and sequencing. Linearized and purified plasmid DNA was *in vitro* transcribed with the RiboMAX Large Scale RNA Production Systems (Promega, Mannheim, Germany) and subsequently a DNase I digestion was performed using the SP6/T7 Transcription Kit (Roche Diagnostics, Mannheim, Germany) according to the manufacturer’s instructions. During purification of the *in vitro* transcribed RNA using the RNeasy Kit (Qiagen, Hilden, Germany) a second on-column DNase I digestion according to the manufacturer’s recommendations was implemented. The exact number of RNA molecules was calculated as described [16] and a log_10_ dilution series (2 × 10^6^ to 2 × 10^-1^ copies/μl) was prepared. RNA was stored until use at -20°C. The analytical sensitivity was evaluated for the SBV-S3 single-target assay, for a SBV-S3 duplex assay with a universal internal control (IC) system according to [17] based on heterologous RNA and for a second SBV-S3 duplex system using the co-amplification of the housekeeping gene beta-actin as internal control [18].

All real-time PCR reactions were carried out in Bio-Rad 96-well PCR plates using a CFX96 quantitative PCR system (Bio-Rad Laboratories Inc., Hercules, USA). All these analyses were performed as technical duplicates and the quantification cycle (C_q_) values listed in the tables represent the mean C_q_-value. In all experiments a qualified number of controls, namely no template controls (NTC), internal controls (IC) and positive controls (PC), were co-amplified.

## Abbreviations

AINOV: Aino virus; AKAV: Akabane virus; BATV: Batai virus; BUNV: Bunyamwera virus; DOUV: Douglas virus; NRIV: Ngari virus; OROV: Oropouche virus; PEAV: Peaton virus; SABOV: Sabo virus; SANV: Sango virus; SATV: Sathuperi virus; SBV: Schmallenberg virus; SHAV: Shamonda virus; SHUV: Shuni virus; SIMV: Simbu virus; THIV: Thimiri virus; TINV: Tinaroo virus; NTC: No template control; IC: Internal control; PC: Positive control; Cq: Quantification cycle; RT-PCR: Reverse transcriptase polymerase chain reaction.

## Competing interests

The authors declare that they have no competing interests.

## Authors’ contributions

MF BH designed the study. MF performed the laboratory experiments and analyzed the data. MF BH KW MB wrote the manuscript. HS, KW performed the cell culture and titer determination. AW constructed the SBV-S3 standard. MF HS KW AW BH MB contributed to final the manuscript preparation. All authors read and approved the final manuscript.
